# Encoding Mie, plasmonic, and diffractive structural colors in the same pixel

**DOI:** 10.1515/nanoph-2023-0254

**Published:** 2023-06-19

**Authors:** Youngji Kim, Jerome K. Hyun

**Affiliations:** Department of Chemistry and Nanoscience, Ewha Womans University, Seoul, 03760, Republic of Korea

**Keywords:** diffraction, Mie resonance, multi-level encryption, plasmonic resonance, structural color

## Abstract

We present a 1D reflective multi-level structural color design that incorporates Mie, plasmonic, and diffractive mechanisms in the same pixel. Comprised of a metallodielectric grating made of TiO_x_ nanowires sandwiched between Ag thin film and Ag substrate, the design can exhibit either a Mie resonance or a localized plasmonic resonance depending on the polarization of incident light, resulting in dramatically different color states. Due to the periodicity, the grating also diffracts light, providing an additional color state. Since diffraction can be turned on or off by the degree of coherence of the incoming light, both Mie and plasmonic colors can be modulated using objective lenses with different numerical apertures. Exploiting the different color generating modes, we encode four layers of information in a pixel array, where each layer is unveiled using a different combination of excitation and imaging settings. These results introduce new possibilities for data encryption, anticounterfeiting, and data storage.

## 1 Introduction

The need for physical tags that can store and display multiple sets of information is increasing with the demand for more sophisticated product logistics and security technologies. The field of structural colors based on nanoresonators has introduced promising features that can address this need. Two distinct types of structural color mechanisms have been of particular interest, namely the Mie-resonant [1, 2] and plasmonic type [3, 4], due to their non-fading colors, ultra-high resolutions, and large gamut coverages.

While Mie and localized surface plasmonic resonances are both consequences of light coupling to confined geometries, the former occurs inside dielectrics whereas the latter occurs on metal surfaces, leading to different sets of optical constraints that define their properties. Plasmonic resonators [5] have been shown to exhibit distinct colors when excited by incident light of different states in the wavelength [6], angle [7], or polarization [8–11]. The ability to sustain resonances between very narrow metallic gaps translates to ultra-high spatial resolution. However, the metal introduces ohmic losses that degrade the color quality. To this end, dielectric Mie resonators have also been extensively explored as structural coloration candidates due to their low loss and ability to support strong resonances [1, 12]. But for dielectrics with moderate refractive indices (*n* ∼ 2), the leakiness of the resonance makes it difficult to achieve highly saturated colors. Approaches to generate saturated colors have included the use of an array to couple to lattice modes [13, 14] or application of a metal shell that improves light confinement [15].

For encryption, plasmonic units [7], [8], [9, 16], [17], [18] or Mie resonances [19–22] have been used to encode two color states in the same pixel by incorporating 2D anisotropy in the unit shape or in the periods of a 2D lattice, and read out by choice of incident light polarization. In all cases, the resonances along the in-plane orthogonal dimensions have been of the same type, which in certain circumstances may impose geometrical constraints. For example, increasing the lengths of the two dimensions in each unit to tune the colors reduces the 2D spatial density of the units, which severely compromises the reflection intensity for both color states.

In this paper, we achieve Mie, plasmonic, and diffractive reflective colors in a 1D nanoresonator grating, each controlled by the period and/or width of the nanoresonator. Both Mie and plasmonic colors are selectable through the polarization and can be modulated by diffractive effects through the numerical aperture (NA) of the objective lens. Purely diffractive colors are also demonstrated using grazing angle illumination. The ability to generate an expanded number of color states in a single design through different excitation and imaging settings can be useful in building more sophisticated identification tags, anticounterfeiting, high-level security labels, and data storage applications. Furthermore, it can also be used to extend the operating modes of dynamic structural colors [23–29].

## 2 Results and discussion

**Figure 1:**
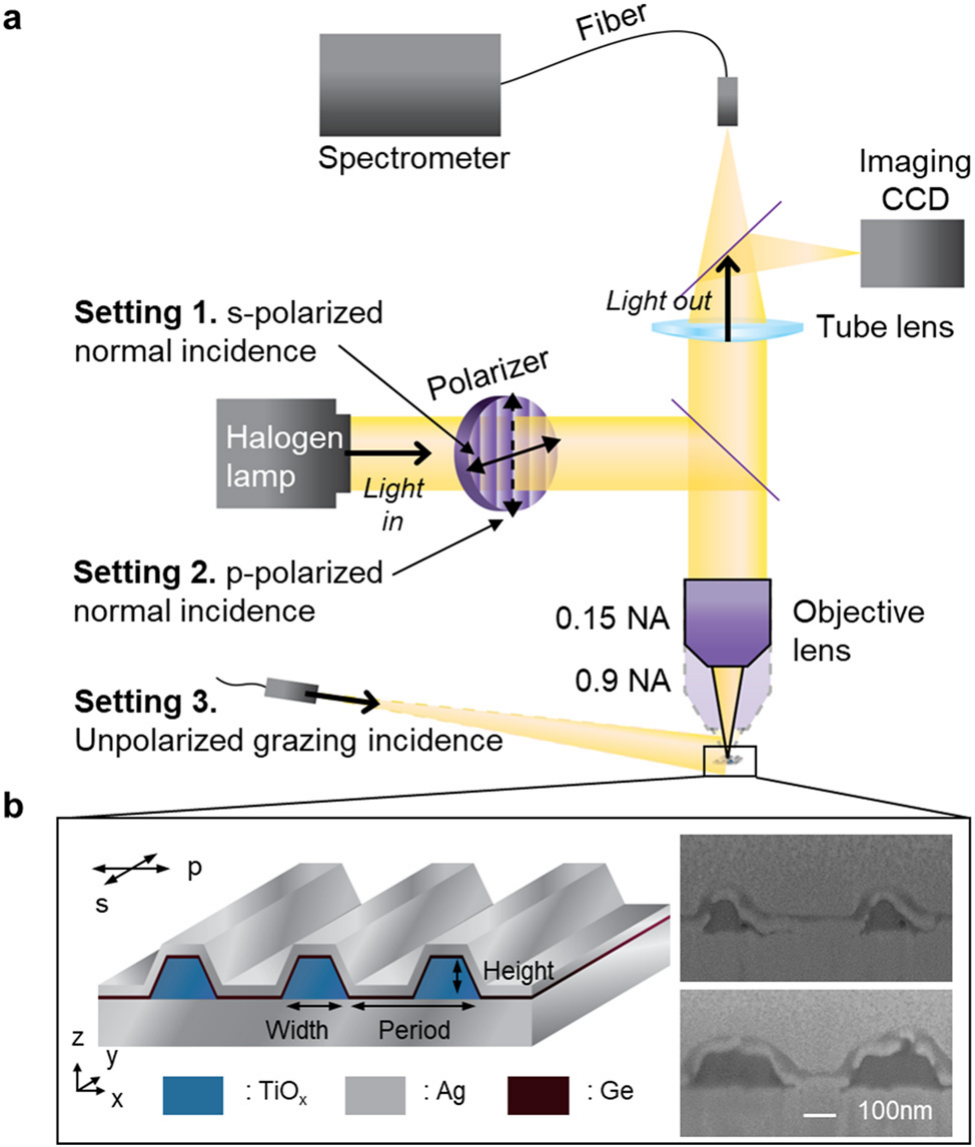
Schematic of the microscopic setup and 1D nanoresonator grating. (a) Schematic of optical measurement setup. For setting 1 (or 2), s- (or p-) polarized white light is injected onto the nanoresonator grating using a linear polarizer. Both injected and reflected light pass through a 0.15 or 0.9 NA objective lens. For setting 3, unpolarized and unfocused white light is injected onto the grating at a grazing angle and the diffracted light is collected through a 0.15 NA objective lens. (b) (left) Schematic of a 1D nanoresonator grating consisting of periodically spaced TiO_x_ nanowires on a Ag substrate embedded under a sub-1nm-thick Ge wetting layer and a 30 nm-thick Ag film. (right) SEM cross-sectioned images of fabricated nanoresonator gratings with a 500 nm period and 147 (top) and 319 nm (bottom) resonator width.

The schematic of the microscopic setup and 1D nanoresonator grating are described in Figure 1a and b, respectively. To keep the optical losses small, _x_ and Ag were used for the dielectric and metal, respectively. A typical pixel consists of periodically spaced TiO_x_ nanowires on a Ag substrate, embedded under a 30 nm-thick Ag film, as illustrated in Figure 1b. A sub 1 nm-thick layer of Ge rests between the TiO_x_ nanowires and Ag film to improve the Ag adhesion and surface roughness [30, 31] and dissipate the highly confined field in the TiO_x_ nanowire more effectively than the Ag shell alone as shown in Figure S1, leading to a pronounced valley in the reflection spectrum that improves the color definition. Each pixel was fabricated by standard e-beam lithography and evaporation methods (Figure S2, see Section 4), resulting in nanowires with trapezoidal cross sections, as shown in the SEM images (Figure 1b, right). The width, herein, refers to the base width of the TiO_x_ nanowire. Three sets of pixels with different periods of 500, 600, and 650 nm were prepared, each sharing widths ranging from 147 to 347 nm and a fixed height of 110 nm.

To investigate the multiple color states of the pixel, brightfield microscopy was operated under different configurations, as illustrated in Figure 1a. For settings 1 and 2, parallel (s-pol) and perpendicular (p-pol) electric fields with respect to the nanowire axis, respectively, were defined by a linear polarizer and directed along the vertical optical axis by a beam splitter for normal injection onto the pixel. The injected and collected light passes through an objective lens whose incident and collection angles were defined by either a 0.15 or 0.9 NA lens. Setting 3 describes grazing angle illumination of unfocused and unpolarized white light from the output of a fiber and the collection of diffracted light with a 0.15 NA lens directly above the grating.

We first describe Mie resonances excited by normally incident s-pol light. We previously demonstrated a similar resonator but in transmission mode [15], where scattering was reduced at the electric dipole Mie resonance due to an opposite dipole moment that forms in the Ag shell [32, 33]. The nanoresonator in our current study operates in reflection, which leads to distinctive scattering (Fano-type) and absorption behavior (Supplementary Note 1 and Figure S3).

**Figure 2:**
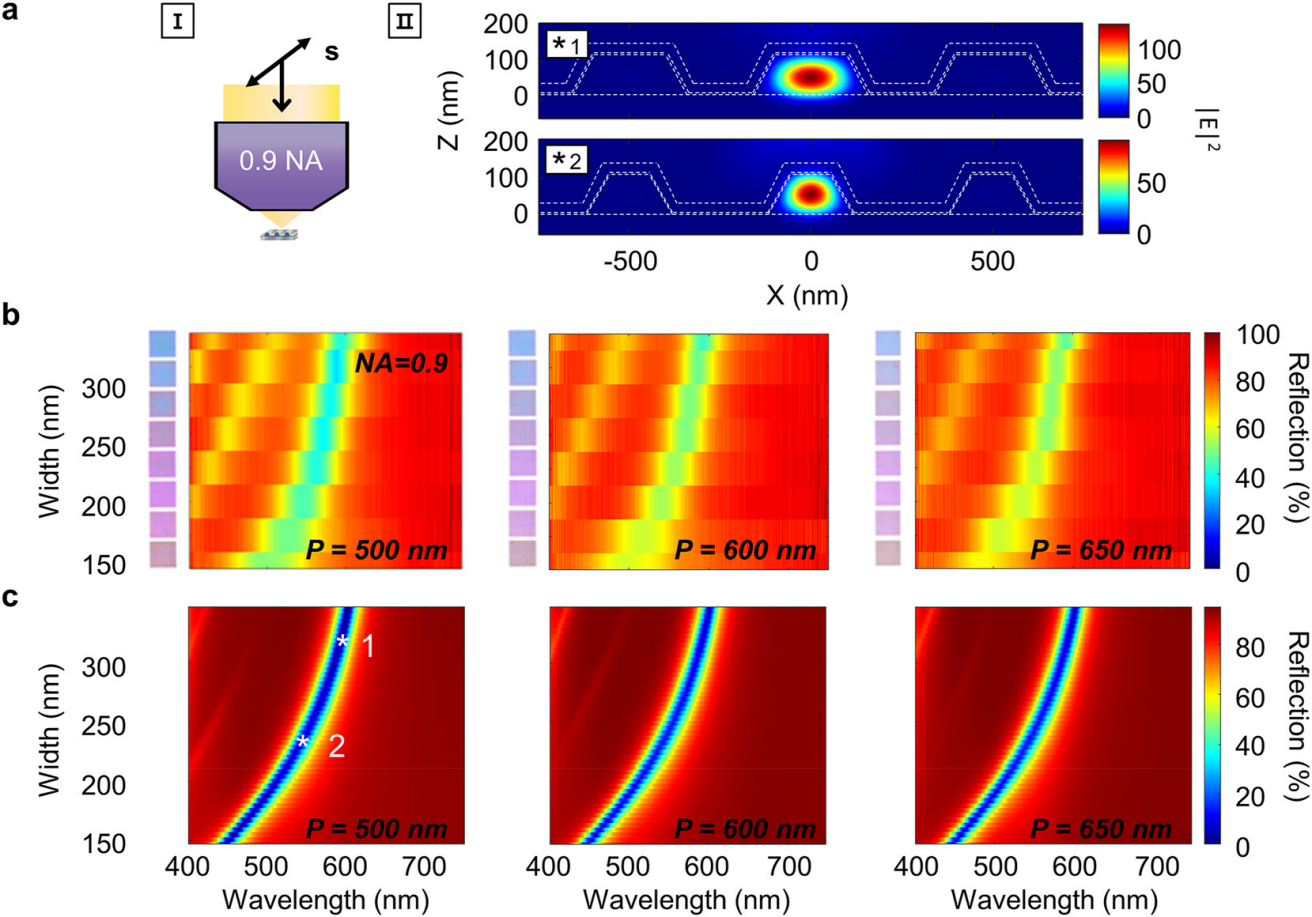
Response of nanoresonator grating to s-polarized incident light (setting 1) through a 0.9 NA objective lens. (a) Schematic of imaging setting (I) and simulated electric field distributions (II) of (*1) 319 nm and (*2) 233 nm-width nanoresonator gratings at the resonance wavelengths of 593 nm and 543 nm, respectively. (b, c) Measured reflection (b) and calculated total reflection (c) spectra as a function of the width and wavelength for periods of 500, 600, and 650 nm. White stars in (c) pinpoint the widths and wavelengths used in (II). Inset of (b) displays images of each pixel obtained with the current imaging setting.

To understand the reflected colors from individual Mie nanoresonators, we viewed the three sets of pixels with a 0.9 NA objective lens. This lens setting focuses light (Figure 2a, left) into a diffraction limited spot smaller than 500 nm for the wavelengths under consideration. This is equal to or less than the smallest investigated period. In fact, finite difference time domain (FDTD) simulations (see Section 4) of a grating with a period of 500 nm show that the focused field is spatially limited to one nanoresonator (Figure 2a, right). Experimentally, the illuminated field of view under the 0.9 NA lens is of high spatial resolution but also of low spatial coherence. This means that the Mie resonances excited in neighboring nanoresonators are not in phase with each other and diffraction is not expected to occur. As a result, the resonance is independent of the period and to a large extent controlled by the width. This is shown more clearly in Figure 2b. The measured reflection spectra for all three periods share similar trends characterized by the fundamental resonance redshifting from 500 to 600 nm with increasing width as expected from the growing resonator size. The corresponding colors that range from pink to light blue with increasing width appear the same for all three periods as shown in the insets, further confirming that any changes in color occur with width rather than period.

To understand the measured reflection, we simulated the total reflection spectra over varying widths for light incident through a 0.9 NA lens (Figure 2c). The total reflection accounts for all scattered light above the resonator and serves as a good measure of light collected by the 0.9 NA lens as the lens has a large acceptance angle of ∼±64°. The simulated results agree well with the measurements and most notably confirm the width-dependence and period-independence of the Mie resonance (see Supplementary Note 2 and Figure S4 for further details). Subtle discrepancies in the intensity between measurement and simulations are detailed in Supplementary Note 3.

**Figure 3:**
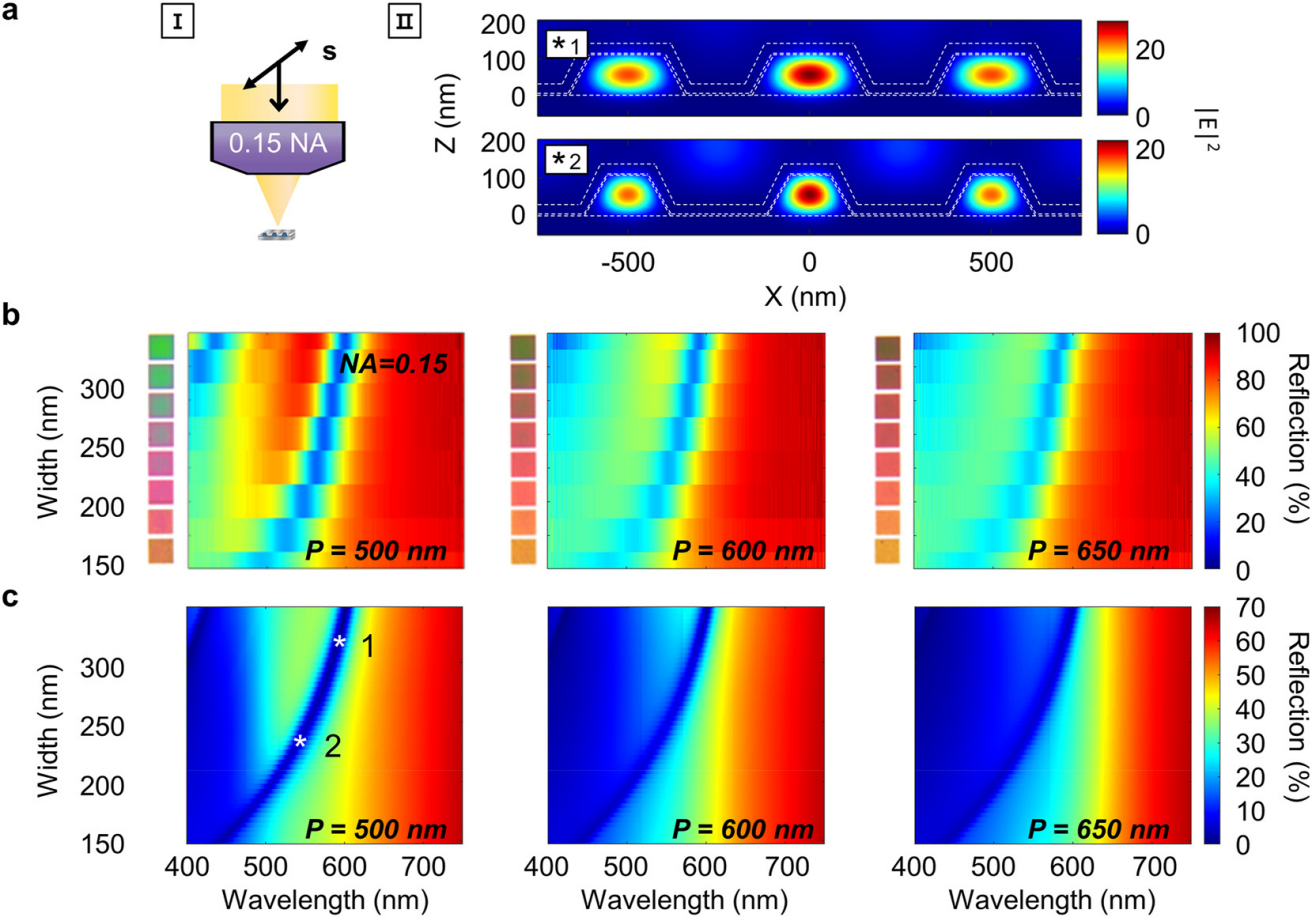
Response of nanoresonator grating to s-polarized incident light (setting 1) through a 0.15 NA objective lens. (a) Schematic of imaging setting (I) and simulated electric field distributions (II) of (*1) 319 nm and (*2) 233 nm-width nanoresonator gratings at the resonance wavelength of 593 nm and 541 nm, respectively. (b, c) Measured reflection (b) and calculated 0th order reflection (c) spectra as a function of the width and wavelength for periods of 500, 600, and 650 nm. White stars in (c) pinpoint the widths and wavelengths used in (a). Inset of (II) displays images of each pixel obtained with the current imaging setting.

We now investigate collectively excited nanoresonators using a 0.15 NA lens. In this case, the diffraction-limited focus is large enough to coherently excite a few resonators (see Figure 3a) and enable diffraction. Here, the collection angle is only ∼±9° such that higher order diffracted orders do not contribute to the spectra, leaving the spectra to be characterized mainly by the 0th order reflection. The resultant spectra display the familiar electric dipole resonances that vary with width, now superimposed on a diffraction-mediated background that varies with period as shown in Figure 3b. The background intensity is significantly reduced for wavelengths much smaller than the period, or the Rayleigh anomaly (RA), due to rejection of the 1st order diffracted light by the objective lens. The intensity transition at the RA is broad across the wavelengths because only a finite number of resonators are coherently excited. This contrasts from the sharp step-like transition induced by an infinite number of resonators excited by a plane wave. The result of the period-dependent spectral modulation is clearly shown in the different shades of color between pixels of the same width but different periods (inset of Figure 3b), which are in stark contrast to the same set of colors observed under the 0.9 NA lens (inset of Figure 2b). We also find that the combined dependence of period and width on the spectra yields more spectral variation that provides a wider range of colors including orange and green than that observed with the 0.9 NA lens. Simulations of the 0th order reflection are in good agreement with the measured trends as shown in Figure 3c, confirming the contributions of both width-dependent Mie resonance and period-dependent 0th order reflection.

We note that while all-dielectric Mie resonators assembled as gratings or arrays can support the coupling between the RA and Mie resonance [34], resulting in so-called surface lattice resonances [35] characterized by Fano profiles [13, 36], we do not observe such an effect in our system. This is presumably because the number of coherently excited nanoresonators is limited to only a few and the Ag film on which they rest is optically lossy.

We now turn our attention to the p-polarized excitation (setting 2). As opposed to the s-pol resonances predominantly formed inside the dielectric, p-pol resonances occur at the interface between the metal and dielectric, representing an inherently distinct mode for color generation (Figure S5). Similar to the s-pol results, p-pol light under a 0.9 NA lens is of low spatial coherence and therefore does not excite any diffractive modes such as surface plasmon polaritons (SPPs) and RAs but mainly excites localized surface plasmons (Figure S5a). We also note that although it is possible to excite magnetic Mie resonances in a 1D nanowire with p-pol light, we do not find strong signatures of such modes in the wavelength range of interest. Additionally, the magnetic Mie resonance would be significantly weak because the Ge layer attenuates the electric fields circulating along the rim of the TiO_x_ core.

Our measured p-pol spectra and colors are of low intensity (Figure S5b). This is because p-pol light incident over a large angular range can excite localized surface plasmonic resonances at more than one site including the Ag shell, the air-Ag interface besides the nanowire, and the base of the TiO_x_ nanowire (Figure S5a and c). Experimentally, the Ag exhibits a degree of surface roughness, which attenuates and dampens many of these resonances. As a result, intensity is suppressed over a few bands, darkening the pixels regardless of nanoresonator width and period.

**Figure 4:**
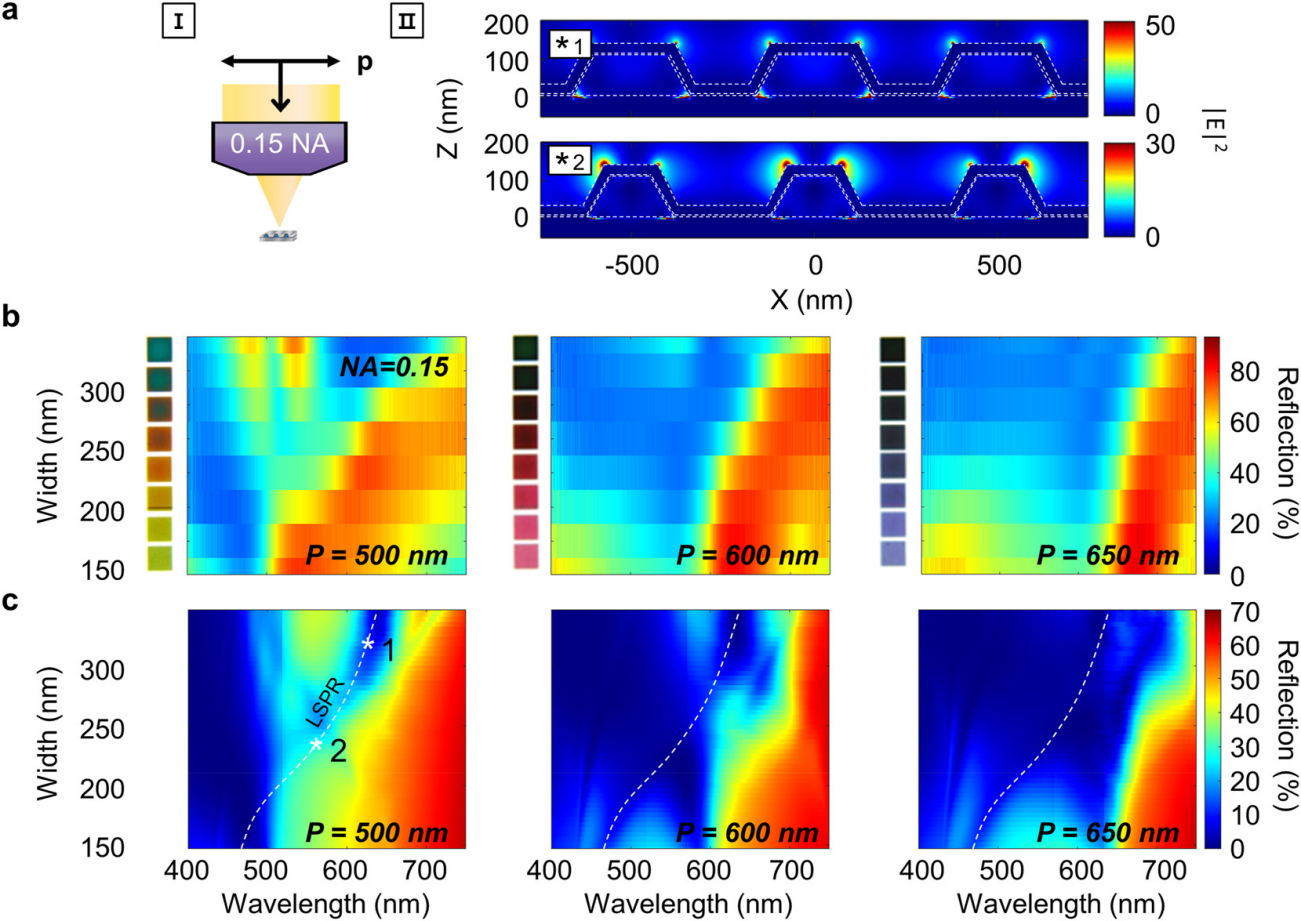
Response of nanoresonator grating to p-polarized incidence light (setting 2) through a 0.15 NA objective lens. (a) Schematic of imaging setting (I) and simulated electric field distributions (II) of (*1) 319 nm and (*2) 233 nm-width nanoresonator gratings at the resonance wavelength of 636 nm and 549 nm, respectively. (b, c) Measured reflection (b) and calculated 0th order reflection (c) spectra as a function of the width and wavelength for periods of 500, 600, and 650 nm. White stars in (c) pinpoint the widths and wavelengths used in (II). Inset of (b) displays images of each pixel obtained with the current imaging setting.

Conversely, use of a 0.15 NA lens greatly simplifies the p-pol reflection spectra, giving rise to vivid colors. By providing a higher degree of coherence, the setting collectively excites a few nanoresonators to generate diffraction (Figure 4a). At the same time, the setting rejects 1st order diffraction due to the narrow collection angle such that little or no intensity exists at wavelengths below the RA. Additionally, the low angular spread of incident light helps limit the number of excitation sites. As a result, the reflection spectra measured over varying widths using a 0.15 NA lens for the three sets of pixels (Figure 4b) show that the observed colors are predominantly composed of intensity from wavelengths above the RA modulated by specific localized surface plasmonic resonances. Below the RA, one can also observe stronger 0th order intensity occurring at smaller widths due to the decreased 1st order diffraction efficiency. These general trends are in agreement with the simulated results (Figure 4c). Finer simulated details that are unresolved in the measurement are described in Supplementary Note 4 and Figure S6.

Localized surface plasmons are characterized by a width dependent valley in the 0th order intensity at wavelengths above the RA, as shown in the simulated spectra. From electric-field distributions, these localized surface plasmons are found to arise at the top two outer corners and bottom two inner corners of the Ag shell (Figure 4a), thereby shifting in wavelength with changes in nanoresonator width and also spatially separating the resonance from the those of neighboring units (see Supplementary Note 5 and Figure S7). The corresponding images of the pixels, shown in the inset, depict bright colors for nanoresonators with smaller widths and darker colors for larger widths. These colors are completely distinct from those observed with s-pol light and are found to change more dramatically with width and period.

**Figure 5:**
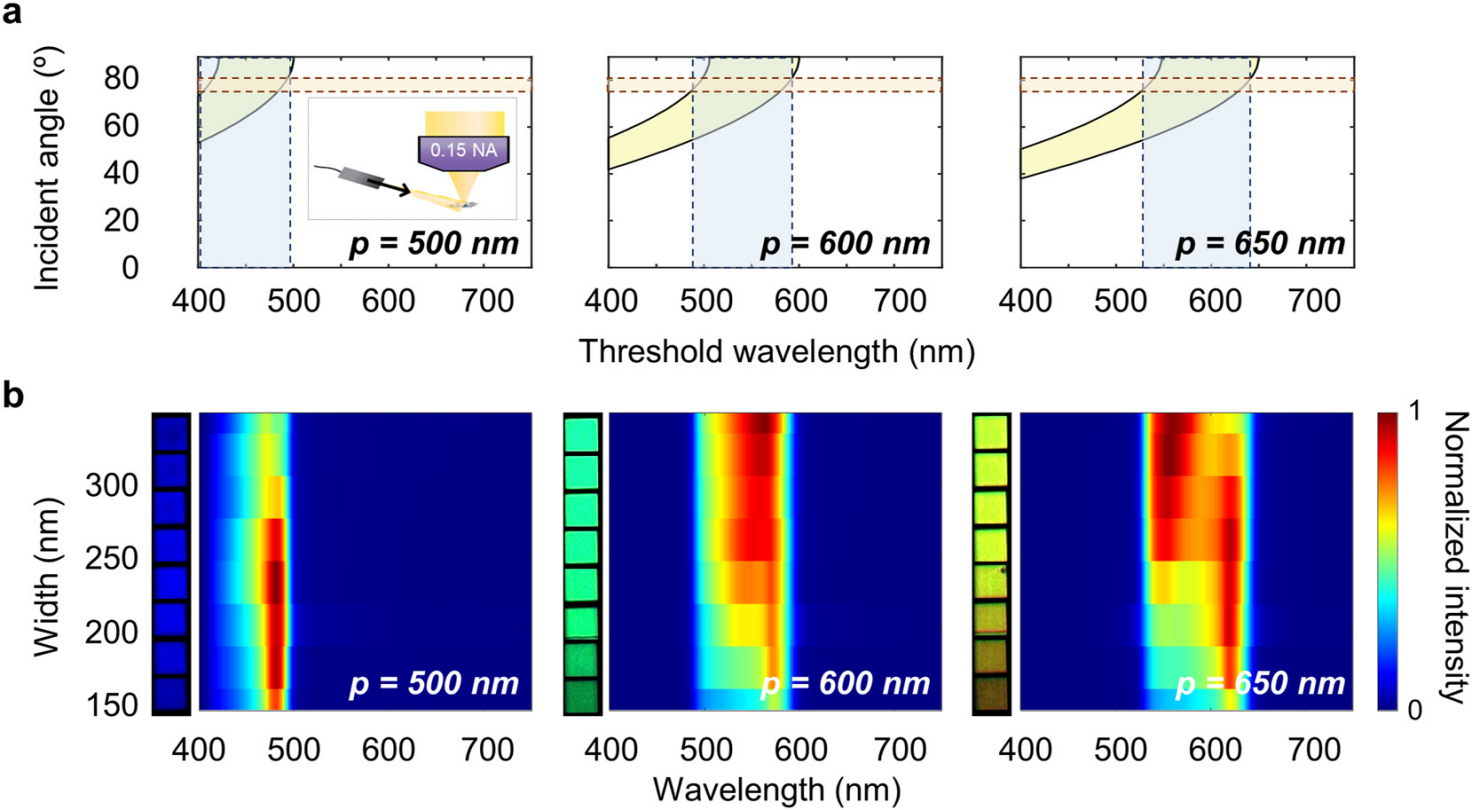
First negative order diffraction of unpolarized light incident at grazing angles by the nanoresonator grating. (a) Relation between the wavelength of diffracted light collected by a 0.15 NA objective lens and angle of incidence for the three different periods of 500, 600, and 650 nm. The yellow area indicates the permissible span of incident angles and associated diffracted wavelengths that can be collected by a 0.15 NA objective lens. The orange shaded area represents the experimental range of incident grazing angles from 75 to 80°. (b) Measured diffraction spectra as a function of width and wavelength for the three different periods of 500, 600, and 650 nm. Inset displays images of each pixel obtained with the current imaging setting.

So far, the described colors depend on either the width or the combination of width and period. To effectively encode and independently control two colors, there must be an additional color generating mode that only depends on the period. This is easily fulfilled by exploiting 1st negative order diffraction (*m* = −1) generated by an angular distribution of off-normal incident light using setting 3 of Figure 1a. Using the diffraction equation (Supplementary Note 6), we plotted in Figure 5a the relation between incident angle and wavelengths of light collected by the 0.15 NA lens for the three sets. Light from the fiber output arrives at the sample over a range of incident angles between 75 and 80°. Shaded in orange, this range corresponds to a collected wavelength span (shaded in blue) of up to 496 nm, 490–590 nm, and 530–645 nm for the 500, 600, and 650 nm-periods, respectively. Our measured 1st negative order diffraction spectra of the three pixel sets further confirm these ranges (Figure 5b), where the measured bandwidths are consistent with the analytically calculated ones. While there are fine spectral details in the measured spectra that capture the complex mixture of plasmonic and dielectric responses from the s and p-polarized fields over the range of incident angles, for color generating purposes the bandwidths and band positions are of greater interest. This is illustrated most clearly by the OM images of pixels with varying widths as shown in the inset of Figure 5b. Despite the different widths, all pixels of the same period display consistent blue, green, and yellow colors for the 500, 600, and 650 nm periods, respectively. We note that although the intensity grows darker for pixels with smaller widths because the diffraction efficiency decreases as the grating volume becomes smaller, the shade of color remains largely similar for every pixel of the same period, indicating that the period rather than the width plays a more dominant role in dictating the color for the respective setting.

**Figure 6:**
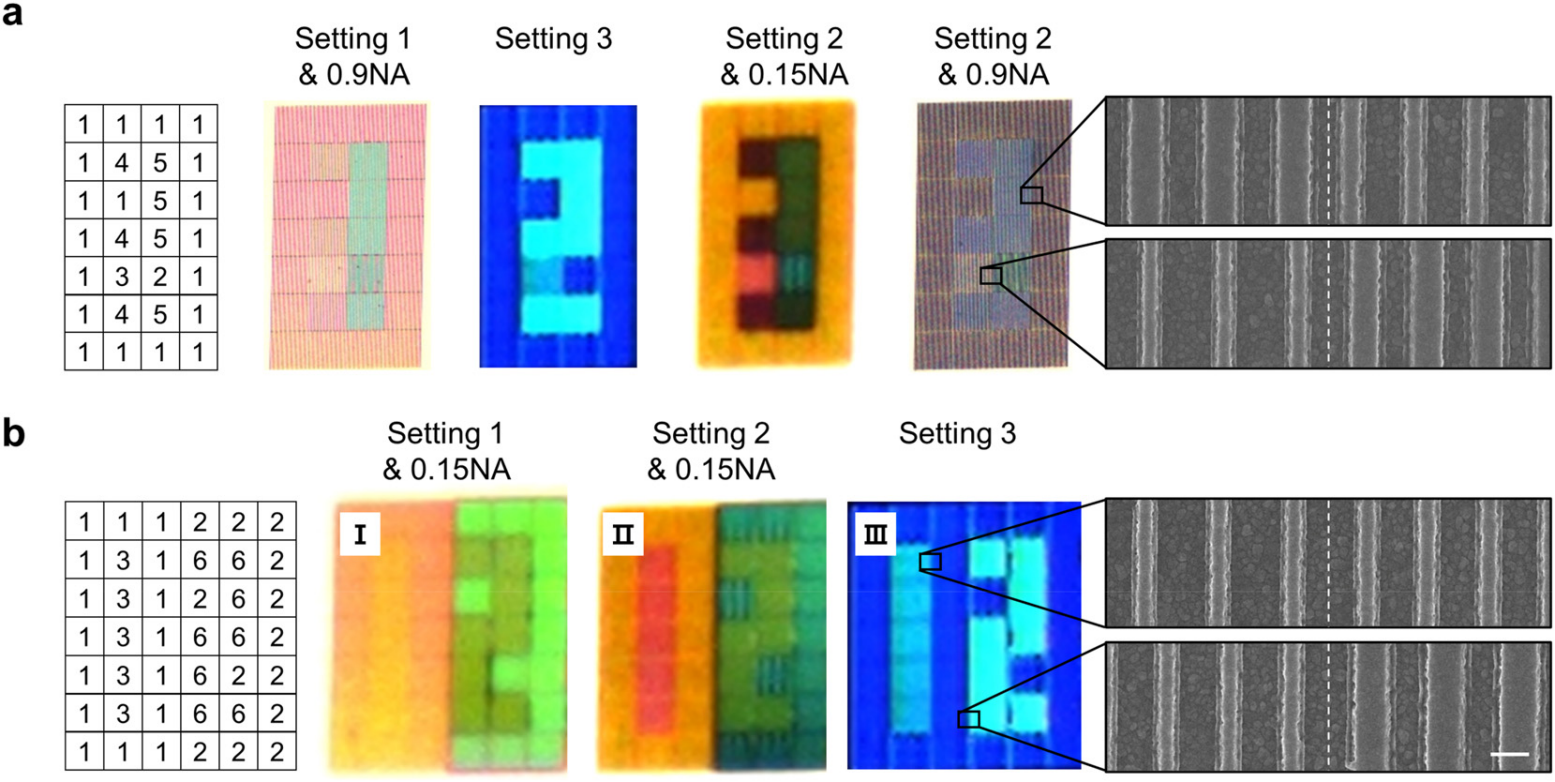
Pixel arrays encoded with multi-level information. (left) Pixel layout with structural parameters detailed in Table 1. (right) SEM images capturing the boundary between structurally different pixels. The nanoresonator width, period, or both are different across the interface (scale bar: 300 nm). (a) Images of the numbers 1, 2, 3, and a blank obtained using setting 1 (s-pol light) with a 0.9 NA lens, setting 3, setting 2 (p-pol light) with a 0.15 NA lens, and setting 2 (p-pol light) with a 0.9 NA lens, respectively. (b) (I) Selective hiding of the number 1 and revealing of the number 2 using setting 1 (s-pol light) with a 0.15 NA lens. (II) Selective revealing of the number 1 and hiding of the number 2 using setting 2 (p-pol light) with a 0.15 NA lens. (III) Selective revealing of both numbers 1 and 2 using setting 3.

The above results show that different colors can be displayed from the same pixel by choice of imaging setting. To see how these results can be used in practice, we demonstrate two simple prints encoded with a few layers of information (Figure 6). These prints were made from an array of pixels, the periods and widths of which are specified in Table 1. SEM images of selected regions portray distinct widths and/or periods across the pixel boundaries (Figure 6a and b right). To uncover each color encoded in the pixel array, imaging settings with different combinations of polarization states (setting 1 and 2) and NAs in addition to setting 3 were used. Setting 1 excites Mie resonant colors with strong width dependence under the 0.9 NA lens and an additional period dependence under the 0.15 NA lens. For setting 2 under a 0.15 NA lens, a whole new set of plasmonic colors that are both dependent on the width and period are excited. Switching the NA to 0.9 causes the colors to darken across all pixels, ideal for creating a blank image. Finally, setting 3 excites a distinct set of diffractive colors dependent on the period.

**Table 1:** Structural parameters of the pixels used in Figure 6.

	1	2	3	4	5	6
Period (nm)	500	500	600	600	600	600
Width (nm)	147	233	147	204	261	290

We first demonstrate a print encoded with four-layers of information consisting of images of the number ‘1’, ‘2’, ‘3’, and a blank (Figure 6a). Our encoding strategy is described in Supplementary Note 7. We can observe the number ‘1’ for 0.9 NA-setting 1 and ‘2’ for setting 3. To introduce a third layer of information, shared or similar colors generated by 0.9 NA-setting 1 and 0.15 NA-setting 2 are first identified from pixels with different widths but of the same period. It is then possible to use the pairs of pixels to blend with neighboring colors in one setting and appears completely different in another. This approach was exploited to produce an image of the number ‘3’ under 0.15 NA-setting 2. The final layer of information is the blank image, which is trivial to produce using 0.9 NA-setting 2. These results also show that for a fixed polarization setting, a change in the NA can dramatically change the colors under p-polarized light. Although not shown, similar scenarios can be achieved with s-pol light, suggesting that NA changes alone may be used as a dedicated mode for color variation and information encryption. Using a similar strategy detailed in Supplementary Note 7, we demonstrate a second example, where the numbers ‘1’ and ‘2’ each appear individually or together depending on the imaging setting. It is worth noting that in all demonstrations, the color variation from polarization switching occurs without the need to change structural dimensions orthogonal to the nanoresonator axis. This helps to minimize changes in the number of nanoresonators within a given pixel area, limiting severe decreases in reflection intensity.

## 3 Conclusions

In summary, we have demonstrated multiple reflective structural colors based on Mie, plasmonic, and diffraction mechanisms stored in a 1D nanoresonator grating. Each mechanism and accompanying color can be selectively activated by choice of light polarization and/or angle of incidence and collection. With s-pol and p-pol light, Mie resonances within the nanoresonator core and LSPRs on the Ag shell can be excited, respectively, giving rise to vibrant and distinct colors. Both resonances are controlled by the nanoresonator width while the background intensity across the wavelengths is controlled by the period for s-pol and p-pol light, the effect of which is more noticeable under low NA lens. Imaging the 1st negative order diffraction from side-illumination introduces another set of colors that are predominantly dependent on the period. By defining the nanoresonator width and period in each pixel of a pixel array, we encoded multiple layers of information where each layer is dominated by a different mechanism. These results offer interesting prospects for applying bright structural colors in sophisticated multi-level security tags and information encryption technologies.

## 4 Methods

### 4.1 Optimization of geometrical parameters

The periods of 500, 600, and 650 nm were selected to ensure the occurrence of the diffraction effect within the visible range. For the Mie and plasmonic colors, the two parameters of importance are the height and width of the nanoresonator as they define the resonance wavelengths. Of these two parameters, the height was fixed to provide resonances within the visible range with experimentally-realizable widths. For a given amount of Ge, we found that, theoretically, a height of 110 nm and the widths of 230–350 nm gave near-zero reflection for resonances in the red.

### 4.2 Pixel fabrication

A sub-1 nm thick Ge wetting layer and ∼200 nm-thick Ag layer were sequentially e-beam evaporated onto a Si wafer. Positive resist (PMMA A4) was spin-coated on top of the Ag layer to a thickness of around 250 nm. E-beam lithography was used to write repeating rows of 10 μm-long grating patterns over a 50 × 50 μm^2^-sized area. The patterns were developed in a 3:1 mixture of IPA and methyl isobutyl ketone (MIBK) for 45 s. A 110 nm-thick layer of TiO_x_ was e-beam evaporated at a 0.04 nm/s deposition rate onto the developed patterns, followed by lift-off. Finally, a sub-1nm thick layer of Ge and 30 nm-thick layer of Ag were sequentially evaporated onto the final patterns.

### 4.3 Calculations

FDTD simulations were performed to compute the reflection, electric field distributions and absorption from the 1D nanoresonator grating. The incident electric field was configured to be parallel or perpendicular to the 1D nanoresonator axis for the s- or p-polarized setting, respectively. To model light through a numerical aperture, a Gaussian source with a thin lens option of 0.15 or 0.9 NA was used while perfectly matched layer (PML) boundary conditions were implemented. For the electric field distribution, 233 and 319 nm-wide nanoresonators were used in the simulation volume for the considered periods of 500, 600 and 650 nm. For the absorption and scattering simulations (Figure S3), a total-field scattered-field (TFSF) source was used. Other simulations (Figures S1, S4, S6, and S7) including the electric field distributions, absorption, and reflection were performed with a planewave light source and periodic boundary conditions for the in-plane dimensions and PML boundary conditions for the out-of-plane dimension.

### 4.4 Optical measurement

Reflection from setting 1 (s-polarized light) and setting 2 (p-polarized light) were measured with a home-built confocal microscope. White light from a halogen lamp was injected onto the pixels through a linear polarizer that rendered the light s (setting 1) or p-polarized (setting 2). Light was focused through a 0.15 or 0. 9NA lens, collected through the same lens, and delivered onto a CCD (STC-TC202USB-AS, SENTECH) or spectrometer (Acton SP2300, Princeton Instruments) to obtain an image or spectrum, respectively. Unfocused light was injected at a grazing angle from the output of a white light fiber from the halogen lamp (HL-2000-HP-FHSA, Ocean Insight) for the diffraction measurement (setting 3). Diffracted light was then collected by a 0.15 NA objective lens and imaged with an imaging camera (INFINITY3-6UR, Teledyne Lumenera) or analyzed with a spectrometer (SpectraPro HRS-300, Teledyne Princeton Instruments).

## Supporting Information

The supporting information is available which includes simulated optical responses of nanoresonator grating without Ge, sample fabrication steps, simulated scattering and absorption cross sections, simulated reflection spectra with varying period under s- and p-pol light, simulated and experimentally obtained optical responses under p-pol normally incident light using a 0.9 NA objective lens, and simulated p-pol reflection spectra of a Ag lamellar grating.

## Supplementary Material

Supplementary Material Details
