# Supplementary material for: Encoding Mie, plasmonic, and diffractive structural colors in the same pixel
Source: Nanophotonics. 2023 Jun 19;12(16):3341–9. doi: 10.1515/nanoph-2023-0254 (PMC11501730; doi:10.1515/nanoph-2023-0254)
Supplement: Supplementary file 1 — Supplementary Material Details [file j_nanoph-2023-0254_suppl_001.pdf]

Supporting Information

for

“Encoding Mie, Plasmonic, and Diffractive Structural Colors in the Same Pixel”

*Youngji Kim, Jerome K. Hyun**

Department of Chemistry and Nanoscience, Ewha Womans University, Seoul, 03760, Republic of Korea

E-mail*: kadam.hyun@ewha.ac.kr

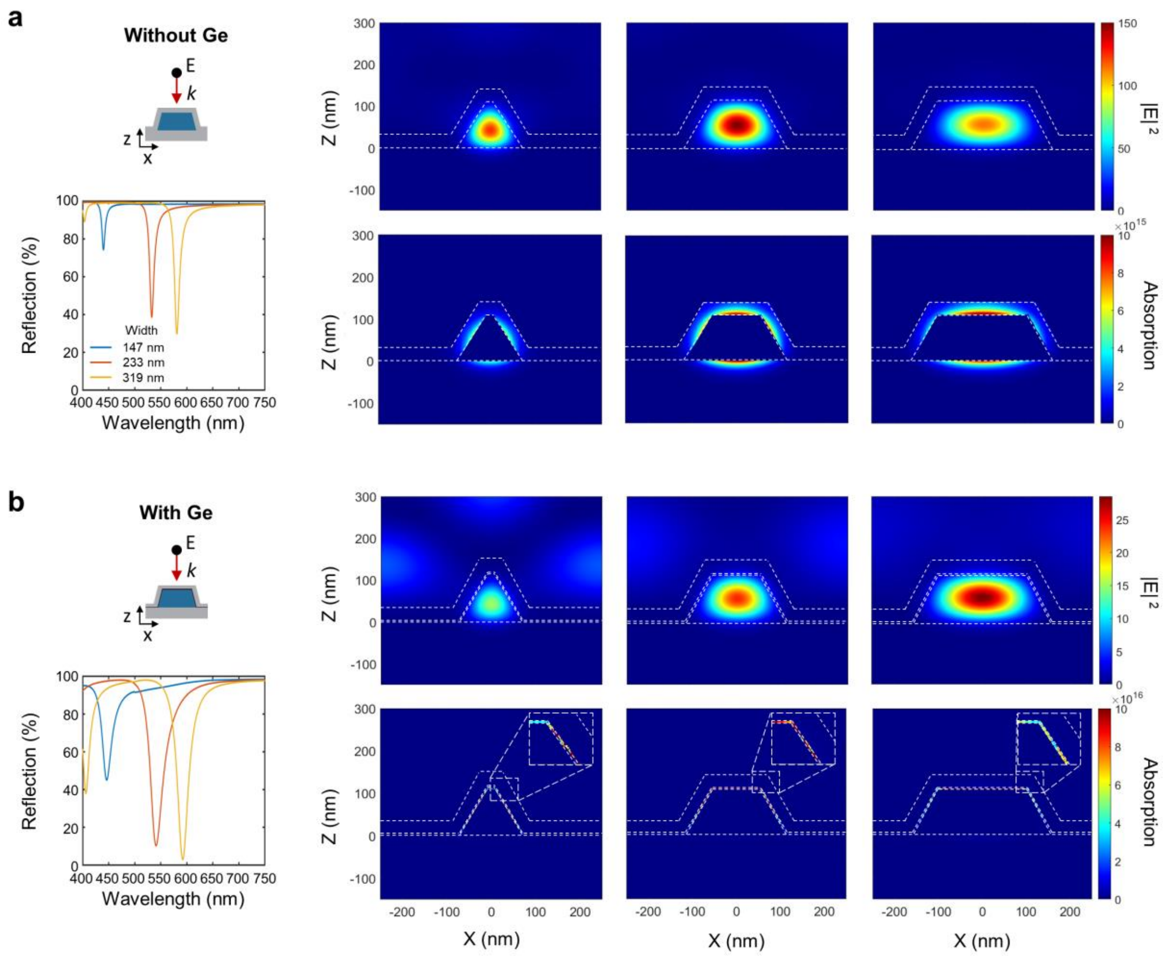

Figure S1. (a, b) Optical responses of nanoresonator grating (a) without and (b) with Ge thin film. (top left) Schematic of grating unit. (bottom left) Calculated total reflection spectra of nanoresonator gratings with 147, 233, and 319 nm-widths and a 500 nm-period. Simulated (top right) electric field and (bottom right) absorption distributions of the grating unit are shown at the resonance wavelengths of 440, 533, and 581 nm without Ge (a), and 446, 541, and 593 nm with Ge (b) for nanoresonators with widths of 147, 233, and 319 nm, respectively, from left to right.

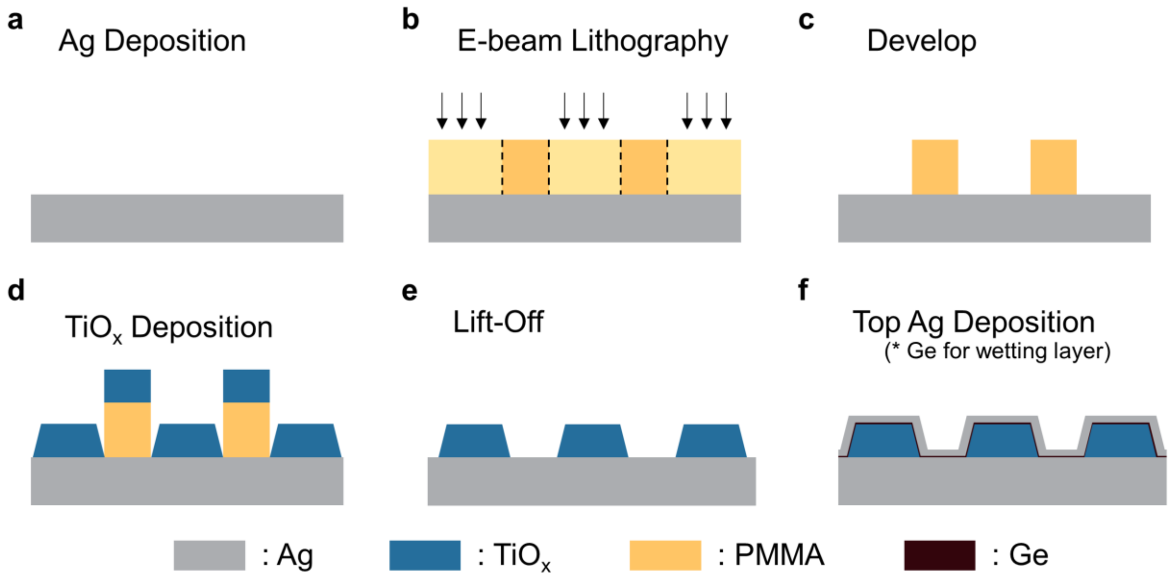

Figure S2. Fabrication process of 1D nanoresonator grating. (a) A sub-1nm thick Ge wetting layer and Ag film with optically opaque thickness are sequentially deposited onto a Si wafer. (b) After spin-coating a layer of PMMA, e-beam lithography is used to write repeating line patterns. (c) The patterns are developed. (d) A 110 nm-thick TiO_x layer is deposited using e-beam evaporation onto the developed patterns. (e) Lift-off process removes the PMMA patterns. (f) A sub-1nm Ge wetting layer and 30 nm-thick Ag layer are sequentially e-beam evaporated.

Supplementary Note 1. Difference between transmissive and reflective Mie nanoresonators

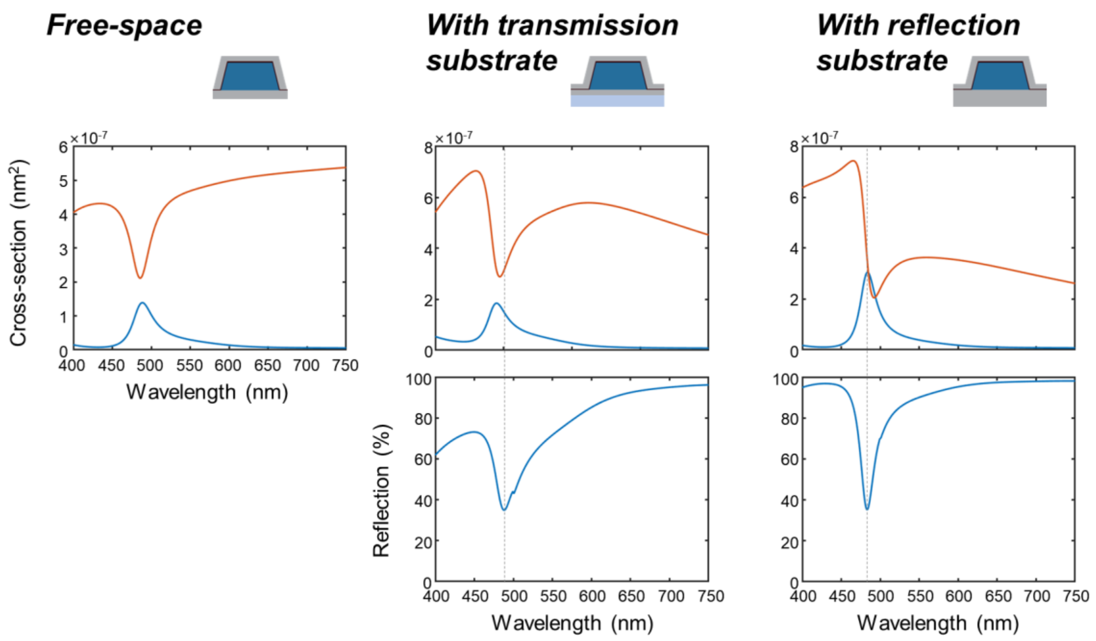

Figure S3. Simulated (top) absorption and scattering cross-sections from a 175 nm-width nanoresonator grating unit in (left) free-space, (middle) on a transmissive substrate, and (right) on a reflective substrate, and (bottom) total reflection of the corresponding nanoresonator grating with a period of 500 nm.

In our previous work, we demonstrated a similar resonator in transmission mode, where scattering was reduced at the electric dipole resonance, enabling light at this wavelength to transmit through the resonator.[1] It was found that the thin Ag film on top of the TiO_x nanowire serves to greatly enhance the resonance by confining the mode and suppress the scattering at the respective wavelength via formation of an opposite dipole moment in the Ag shell as shown in previous studies.[2, 3] Although the Mie nanoresonator of our previous work and the one from our current study share geometrical similarities, a key distinction is that the current nanoresonator uses a reflective substrate rather than a transparent one. This difference, while

subtle, leads to distinctive scattering and absorption behavior. Understanding the absorption peak is particularly important for reflective pixels since it defines the valley in an otherwise featureless reflection spectrum, forming the basis of subtractive color. In the case of the transmissive nanoresonator, the scattering minimum occurs close to the absorption peak position. However, for the reflective nanoresonator, the scattering minimum and absorption peak are further shifted apart from each other. This is attributed to the electric dipole resonance interfering with the reflected light, causing a Fano-like profile[4] in the scattering spectrum as depicted in Figure S3. Characteristic of the Fano line shape, the scattering cross section near the resonance falls sharply with increasing wavelengths, intersecting the absorption cross section before reaching its minimum value. This point at which the absorption and scattering cross sections are equal is known as critical coupling[5] and marks the point of peak absorption. As a result, absorption is peaked at a lower wavelength than the point of minimum scattering.

Supplementary Note 2. Demonstration of width-dependence and period-independence of the Mie resonance

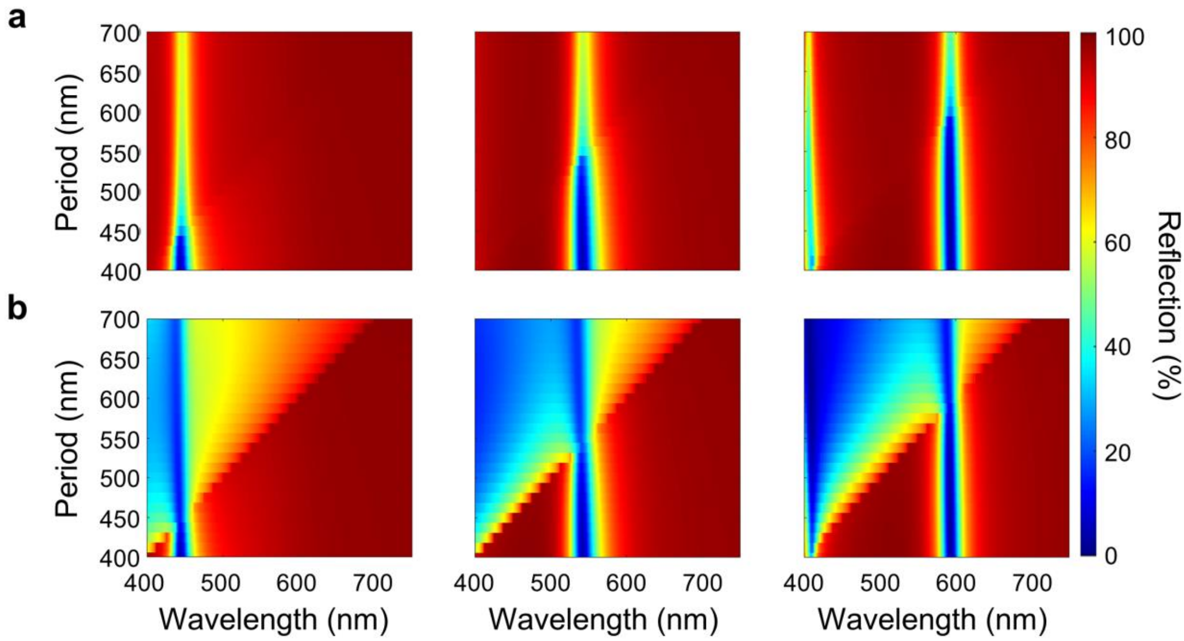

Figure S4. (a, b) Simulated (a) total and (b) 0th order reflection under s-polarized light (setting 1) as a function of period and wavelength for widths of 147, 233, and 319 nm. The simulation was performed with a planewave light source. The resonance wavelength does not shift in wavelength across all periods.

To further verify that the resonance wavelength is independent of the period, we simulated the total reflection spectra for nanoresonators by fixing the width and varying the periods (Figure S4a). For each width, the electric dipole resonance does not shift in wavelength over all periods, indicating that the pixel colors are not affected by the period for the given imaging conditions.

Supplementary Note 3. Details of subtle discrepancies in the intensity between measurement and simulations

We note that although the simulated spectra show consistent intensities for all three periods (Figure 2c), the measured results reveal decreasing valley depths with increasing periods (Figure 2b), where an average reflection value of 47% for the 500 nm-period valley increases to 54% for the 650 nm-period. This is because the simulation treats only one excited nanoresonator and disregards the out-of-phase light coupling to different nanoresonators in the grating. In contrast, our measurement detects the intensities from all resonances within the field of view, the number of which decreases with increasing period. As less nanoresonators absorb within the given pixel area, the valley depth decreases. We note that such change, however, is much smaller than what is observed for polarization dependent plasmonic resonator arrays exploiting near-field coupling between neighboring elements as their colors depend very sensitively on the separation distance.[6]

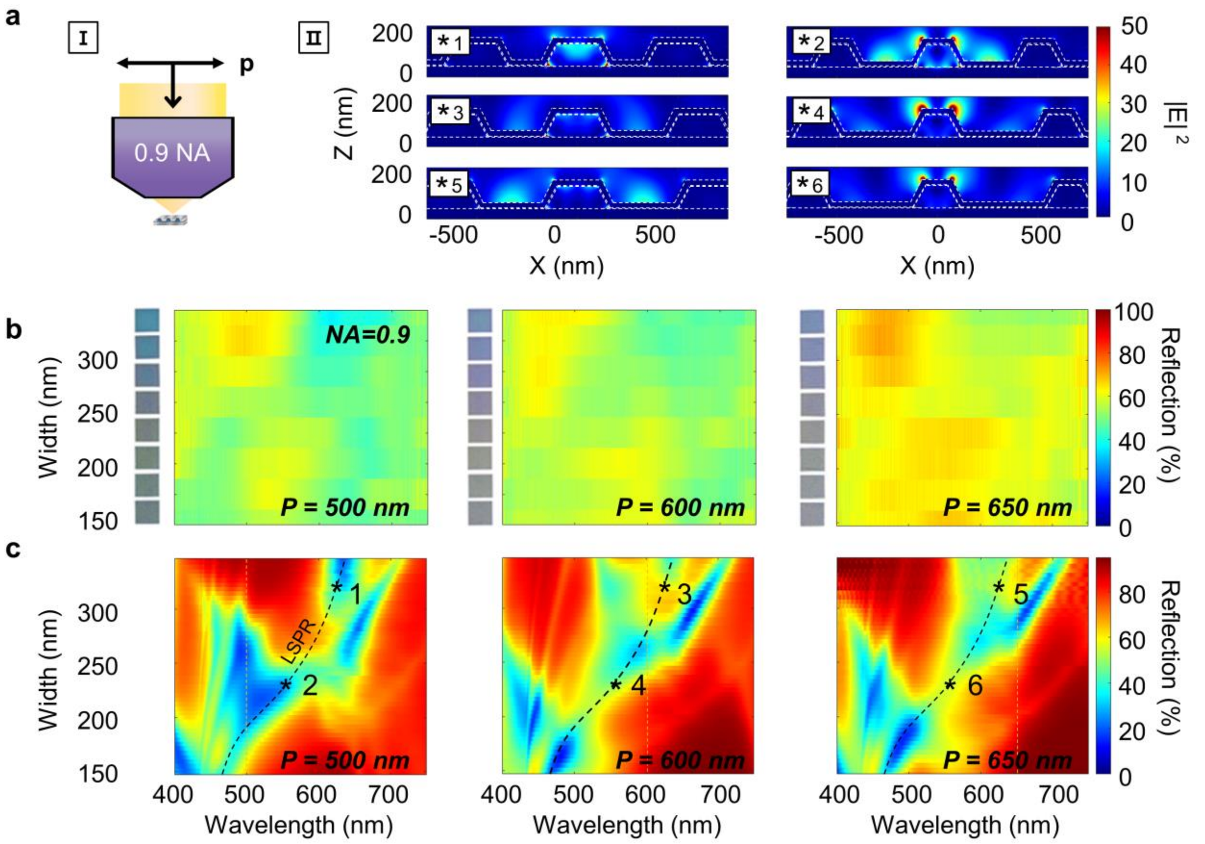

Figure S5. Response of nanoresonator grating to p-polarized incidence light (setting 2) through a 0.9 NA objective lens. (a) Schematic of imaging setting (I) and simulated electric field distributions (II) of (*1, *3, *5) 319 nm and (*2, *4, *6) 233 nm-width nanoresonator gratings with (*1, *2) 500, (*3, *4) 600, and (*5, *6) 650 nm periods at the resonance wavelengths of 636 nm for the 319 nm-width and 549 nm for the 233-width, respectively. (b, c) Measured reflection (b) and calculated total reflection (c) spectra as a function of width and wavelength for periods of 500, 600, and 650 nm. Black stars in (c) pinpoint the widths and wavelengths used in (II). Inset of (b) displays images of each pixel obtained with the current imaging setting.

Supplementary Note 4. Comparison of plasmonic fields between nanoresonator grating and Ag lamellar grating

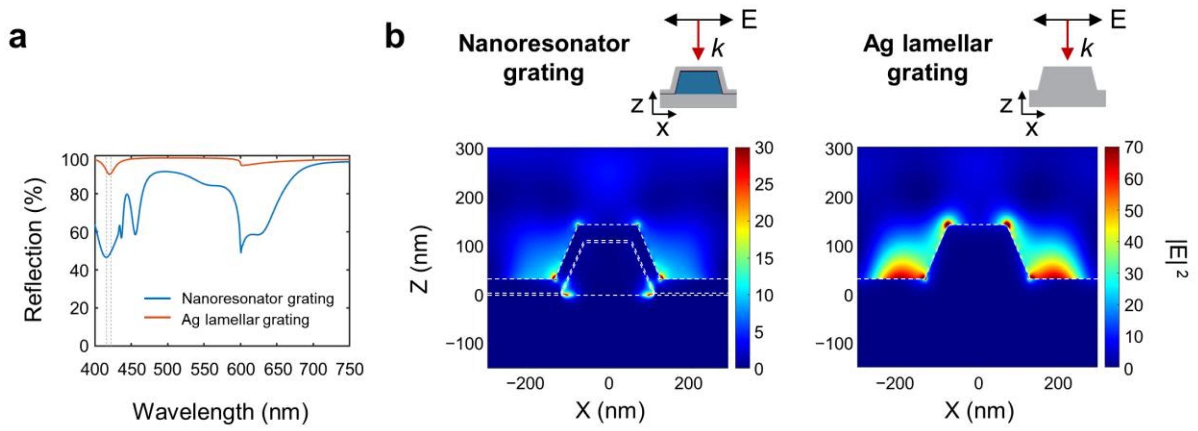

Figure S6. (a) Simulated total reflection spectra of a nanoresonator grating and (b) electric field distributions of a nanoresonator grating unit and Ag lamellar grating unit at the plasmonic resonance wavelengths of 415 and 420 nm, respectively. The simulations were performed with p-polarized normally incident light. SPP fields occurring at the Ag-air interface are observed for both cases.

It is worth noting one fine spectral detail in the simulated results that is unresolved in our measurements due to surface roughness and irregularities. Close inspection at wavelengths between 400 and 500 nm and periods above 600 nm reveals the spectral signature of the 1st SPP branch, where electric field distributions illustrate the characteristic SPP fields occurring at the Ag-air interface adjacent to the nanoresonator (Figure S6a), which is a general phenomenon for plasmonic gratings as illustrated with a simple Ag lamellar grating in Figure S6b.

Supplementary Note 5. Simulated p-pol reflection spectra for fixed widths

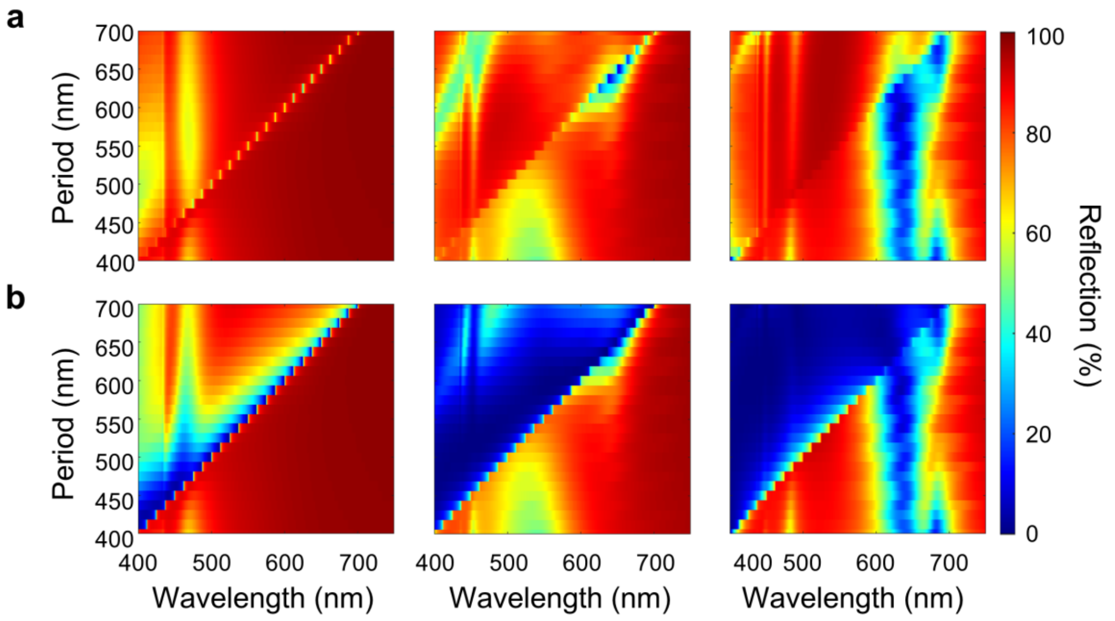

Figure S7. (a, b) Simulated (a) total and (b) 0th order reflection spectra under p-polarized light (setting 2) as a function of period and wavelength for widths of 147, 233, and 319 nm. The simulation was performed with a planewave light source.

One common effect observed with other plasmonic structural colors is the near-field coupling between neighboring units that gives rise to spectral modulations with separation distance. While the near-field coupling can be used as a color tuning feature, it is often nonintuitive to predict. To see if this effect is also present in our nanoresonators, we calculated the reflection spectra over varying periods using a planewave source. Figure S7 shows that the resonance wavelength of the localized surface plasmon from a nanoresonator with fixed width remains unchanged despite changes in the period, confirming that it is largely free from the influence of neighboring modes. This is attributed to the trapezoidal shape of the nanoresonator cross section where the plasmonic sites on the top of the Ag shell are too far removed from equivalent neighboring sites even when the bases of the respective two resonators are slightly touching.

This feature helps prevent spectral shifts of the localized plasmon resonance with period and facilitates prediction of accessible colors.

Supplementary Note 6. Diffraction equation

The relation between the incidence angle, grating period, and wavelength of the diffracted light collected by the objective lens can be found from the diffraction equation:

$$m \times \lambda = P \times (\sin\theta_{in} - \sin\theta_{out}) \quad (1),$$

where m, λ, P, and θ are the diffraction order, diffracted wavelength, period, and incident or diffracted angle with respect to the surface normal, respectively.

Supplementary Note 7. Design strategy for image encryption

Our encoding method takes advantage of the fact that the colors from s-pol light under a 0.9 NA lens are highly dependent on the nanoresonator width while the colors from side-illuminated 1st negative order diffraction are predominantly determined by the period. This dependence of separate imaging modes on distinct spatial parameters allows two independent sets of images to be stored in the same pixel array by adjusting the nanoresonator widths for one image and periods for the other.

Example 1 (Figure 6a). The pixels labelled 4 in the array layout share similar Mie-resonant colors with the pink background under the s-pol 0.9 NA setting, but their plasmonic color, green, is noticeably distinct from the orange background under the p-pol 0.15 NA setting. Because the pixels share the same period, their colors switch to display the number '2' image under the 1st negative order diffraction imaging setting, identical to the way the number '1' image changes to '2'.

Example 2 (Figure 6b). The pixels labelled 3 in the array layout blend with the pink background in image I under the s-pol light but become distinct (pink vs orange) in image II under p-pol light. Similarly, the pixels labelled 6 share a similar dark green color with the background in image II under p-pol light but stand out (olive vs light green) in image I under s-pol light. This allows one to selectively see either the number '1' or '2' while hiding the other number by choice of polarization. Since both numbers are made from pixels having the same period but different from those of the background, switching to the 1st negative order diffraction imaging setting turns on both numbers (cyan numbers vs blue background) as shown in image III.

References

[1] Lee, J. S., Park, J. Y., Kim, Y. H., et al., "Ultrahigh resolution and color gamut with scattering-reducing transmissive pixels," *Nat. Commun.*, vol. 10, no. 1, pp. 4782, 2019.
[2] Alù, A. and Engheta, N., "Achieving transparency with plasmonic and metamaterial coatings," *Physical Review E*, vol. 72, no. 1, pp. 016623, 2005.
[3] Fan, P., Chettiar, U. K., Cao, L., Afshinmanesh, F., Engheta, N. and Brongersma, M. L., "An invisible metal–semiconductor photodetector," *Nat. Photonics*, vol. 6, no. 6, pp. 380-385, 2012.
[4] Liu, T., Xu, R., Yu, P., Wang, Z. and Takahara, J., "Multipole and multimode engineering in Mie resonance-based metastructures," *Nanophotonics*, vol. 9, no. 5, pp. 1115-1137, 2020.
[5] Alaee, R., Albooyeh, M. and Rockstuhl, C., "Theory of metasurface based perfect absorbers," *J. Phys. D: Appl. Phys.*, vol. 50, no. 50, pp. 503002, 2017.
[6] Li, Z., Clark, A. W. and Cooper, J. M., "Dual Color Plasmonic Pixels Create a Polarization Controlled Nano Color Palette," *ACS Nano*, vol. 10, no. 1, pp. 492-498, 2016.
